# Supplementary figures and images for: Toxoplasma gondii harbors a hypoxia-responsive coproporphyrinogen dehydrogenase-like protein
Source: mSphere. 2024 Feb 27;9(3):e00092-24. doi: 10.1128/msphere.00092-24 (PMC10964404; doi:10.1128/msphere.00092-24)

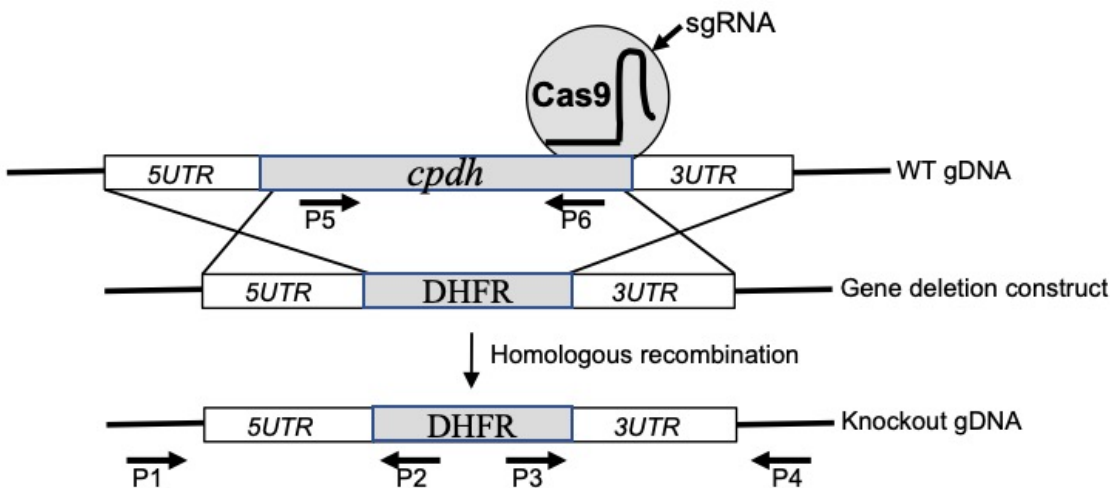

*RHΔku80::nLuc*

*Δcpdh::nLuc*

*ΔcpoxΔcpdh::nLuc*

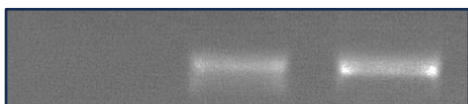

5'ARM, ~380 bp (P1+P2)

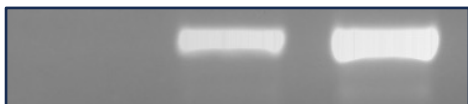

3'ARM, ~410 bp (P3+P4)

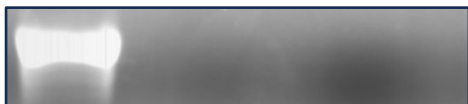

CDS, ~910 bp (P5+P6)

Supplement: Figure S1 — Creation of ∆cpdh::nLuc and ∆cpox∆cpdh::nLuc Toxoplasma strains. [file msphere.00092-24-s0001.pdf]

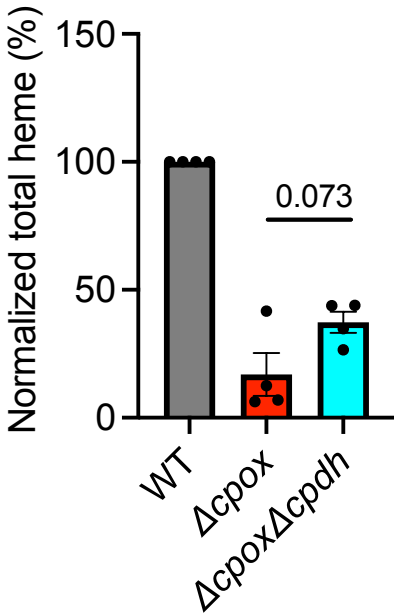

Supplement: Figure S2 — Heme quantification in the TgCPDH-deficient Toxoplasma strain. [file msphere.00092-24-s0002.pdf]
