# Supplementary material for: Toxoplasma gondii harbors a hypoxia-responsive coproporphyrinogen dehydrogenase-like protein
Source: mSphere. 2024 Feb 27;9(3):e00092-24. doi: 10.1128/msphere.00092-24 (PMC10964404; doi:10.1128/msphere.00092-24)
Supplement: Supplemental legends — Legends for supplemental figures and tables. [file msphere.00092-24-s0003.pdf]

SUPPLEMENTAL MATERIAL LEGENDS

Figure S1. Creation of Δ*cpdh::nLuc* and Δ*cpox*Δ*cpdh::nLuc* *Toxoplasma* strains.

Schematic representation of the standard CRISPR-Cas9-based methodology employed for deleting the *TgCPDH* gene in *Toxoplasma*. The successful integration of the pyrimethamine resistance cassette (DHFR) into the *TgCPDH* locus during gene deletion was verified using PCR. The genomic positions of the primers used for PCR are highlighted within the diagram.

Figure S2. Heme quantification in the *TgCPDH*-deficient *Toxoplasma* strain.

The total heme abundance was quantified using a protoporphyrin IX-based fluorescence assay. The WT and Δ*cpox* strains were included for comparison. The total heme levels in Δ*cpox* and Δ*cpox*Δ*cpdh* were normalized against the WT strain. Data were derived from 4 biological replicates with 3 technical replicates each. Statistical significance of the total heme contents between Δ*cpox* and Δ*cpox*Δ*cpdh* was calculated and labeled in the plot by a two-tailed unpaired Student's *t*-test.

Table S1. Primers used in this work.

Table S2. *Toxoplasma* strains used in this work.
